# Somatosensory-Evoked Potentials as a Marker of Functional Neuroplasticity in Athletes: A Systematic Review

**DOI:** 10.3389/fphys.2021.821605

**Published:** 2022-01-17

**Authors:** Tom Maudrich, Susanne Hähner, Rouven Kenville, Patrick Ragert

**Affiliations:** ^1^Department of Movement Neuroscience, Faculty of Sport Science, Leipzig University, Leipzig, Germany; ^2^Department of Neurology, Max Planck Institute for Human Cognitive and Brain Sciences, Leipzig, Germany

**Keywords:** athletes, somatosensory-evoked potential, neuroplasticity, sensory processing, systematic review

## Abstract

**Background:**

Somatosensory-evoked potentials (SEP) represent a non-invasive tool to assess neural responses elicited by somatosensory stimuli acquired via electrophysiological recordings. To date, there is no comprehensive evaluation of SEPs for the diagnostic investigation of exercise-induced functional neuroplasticity. This systematic review aims at highlighting the potential of SEP measurements as a diagnostic tool to investigate exercise-induced functional neuroplasticity of the sensorimotor system by reviewing studies comparing SEP parameters between athletes and healthy controls who are not involved in organized sports as well as between athlete cohorts of different sport disciplines.

**Methods:**

A systematic literature search was conducted across three electronic databases (PubMed, Web of Science, and SPORTDiscus) by two independent researchers. Three hundred and ninety-seven records were identified, of which 10 cross-sectional studies were considered eligible.

**Results:**

Differences in SEP amplitudes and latencies between athletes and healthy controls or between athletes of different cohorts as well as associations between SEP parameters and demographic/behavioral variables (years of training, hours of training per week & reaction time) were observed in seven out of 10 included studies. In particular, several studies highlight differences in short- and long-latency SEP parameters, as well as high-frequency oscillations (HFO) when comparing athletes and healthy controls. Neuroplastic differences in athletes appear to be modality-specific as well as dependent on training regimens and sport-specific requirements. This is exemplified by differences in SEP parameters of various athlete populations after stimulation of their primarily trained limb.

**Conclusion:**

Taken together, the existing literature suggests that athletes show specific functional neuroplasticity in the somatosensory system. Therefore, this systematic review highlights the potential of SEP measurements as an easy-to-use and inexpensive diagnostic tool to investigate functional neuroplasticity in the sensorimotor system of athletes. However, there are limitations regarding the small sample sizes and inconsistent methodology of SEP measurements in the studies reviewed. Therefore, future intervention studies are needed to verify and extend the conclusions drawn here.

## Introduction

Exercise induces widespread adaptations in the human body. Such adaptations lead to strong health benefits, especially reduced all-cause mortality, reduced (co-) morbidity, and improvement in overall well-being (Kokkinos, 2012). Among the systems that adapt positively to exercise is the central nervous system (CNS) (Mandolesi et al., 2018). To date, numerous structural and functional changes in the brain and spinal cord have been reported in response to exercise over the lifespan (Valkenborghs et al., 2019; Chen et al., 2020), highlighting its potential to shape the brain and spinal cord in a use-dependent manner.

In recent years, movement neuroscience research has increasingly focused on athletes as their advanced physical abilities are linked to specific CNS adaptations (Nakata et al., 2010). Investigating athletes is of particular interest because organized training, typically characterized by extensive and sustained integration of sensory information to fine-tune motor control strategies, can serve as a robust model for functional and structural neuroplasticity (Yarrow et al., 2009). To date, it remains incompletely understood which physiological and specifically which neurophysiological markers underlie peak athletic performance. Accordingly, novel approaches have been proposed for neurodiagnostic in sports, using neurostimulation methods such as transcranial magnetic stimulation (TMS) (Turco and Nelson, 2021) as well as imaging methods such as magnetic resonance imaging (MRI), electroencephalography (EEG), and functional near-infrared spectroscopy (fNIRS) to identify markers of training-induced neuroplasticity in athletes (Seidel-Marzi and Ragert, 2020).

Another method that has received comparably less attention in the context of neurodiagnostic in sports is the application of somatosensory-evoked potentials (SEP). SEPs represent neural responses elicited by external somatosensory stimuli recorded via electrophysiological methods (Macerollo et al., 2018). These responses reflect the summated electrical activity of postsynaptic potentials from the activation of neural structures along the somatosensory pathway (Cohen, 2017). This technique is a non-invasive method, generally used to investigate the functional organization, integrity, and neuroplasticity of the somatosensory system in humans (Passmore et al., 2014). Its advantage is the relative inexpensiveness and ease of application in comparison with other neuroimaging methods (Macerollo et al., 2018). Most commonly, electrical stimulation of a peripheral nerve, i.e., median nerve and tibial nerve, is applied to examine upper and lower limb evoked responses, respectively. Evoked responses are recorded time-locked and averaged over several trials using EEG with different latencies from the onset of peripheral stimulation. This is due to the sequential excitation of neural generators along the somatosensory pathway through the ascending volley induced by peripheral stimulation (Aminoff and Eisen, 2012). In the SEP waveform nomenclature, the recorded wave is specified by polarity, through the letters N (negative peak) or P (positive peak), followed by an integer, corresponding to the nominal post-stimulus latency (ms). For example, a typically observed negative deflection occurring 20 ms after stimulus onset is denoted as N20. The potentials are identified by their characteristic distribution, which reflects the activation of their generators, and are evaluated in terms of latency (ms), amplitude (V), and inter-peak intervals (Cruccu et al., 2008). Far-field potentials (so-called because their neuronal origin lies far away from the measured EEG electrode) are thought to reflect the peripheral excitation of nerve cells in the spinal cord and subcortical structures (Ghigo et al., 1991; Dumitru and King, 1993). On the contrary, near-field potentials are generated cortically, close to the recording electrode on the scalp. Early, or short-latency cortical SEPs to upper-limb stimulation have peak latencies in the 18–35 ms range. The initial negative deflection which can be observed using EEG over parietal regions is referred to as the N20 component (Yamada, 1988). The N20 component is considered to be generated by neurons in the anterior wall of the postcentral gyrus (S1), Brodmann area (BA) 3b (Allison et al., 1991; Peterson et al., 1995). Additionally, long-latency responses, which likely correspond to higher-order processing of sensory input, can be recorded with maxima at different cortical sites, e.g., P100, bilateral secondary somatosensory cortex (Hari et al., 1993); N140, bilateral frontal lobes involving orbitofrontal, lateral and mesial cortex (Allison et al., 1992); N300, frontal/posterior association cortex and temporal-parietal connection (Valeriani et al., 2001). In the case of tibial nerve stimulation, corresponding latencies of SEP components induced by median nerve stimulation (e.g., N20 reflecting BA 3b activation) prolong (i.e., P37) due to the more distal stimulation site and resulting longer conduction times from nerve to the cortex (Hari et al., 1996). Accordingly, N45 and P65 reflect the activation of BA 1 in S1 in response to tibial nerve stimulation (Kakigi et al., 1995). Finally, high-frequency oscillations (HFO) can be observed in SEPs, typically superimposed on the N20 component at a frequency of ~400–600 Hz (Aminoff and Eisen, 2012; Yamada, 2014). These high-frequency oscillations seem to be generated intracortically by postsynaptic inhibitory interneurons of the primary somatosensory cortex (Urasaki et al., 2002; Gobbelé et al., 2003; Ozaki and Hashimoto, 2011). Taken together, changes in SEPs parameters, both in the time domain and frequency domain are needed to fully understand the location of its generation and how these generators get modulated by specific experimental procedures or interventions (Macerollo et al., 2018).

Traditionally, SEPs have been used as a clinical tool to assess the integrity of both the central and peripheral nervous systems (Walsh et al., 2005). Abnormal values of the SEP may be useful in demonstrating the presence of a lesion in the somatosensory pathways, assisting in its localization, and providing a prognostic guide (Cruccu et al., 2008). In this regard, general guidelines for SEP applications have been formulated by the International Federation of Clinical Neurophysiology (IFCN) (Nuwer et al., 1994; Cruccu et al., 2008). Besides its use in clinical practice, assessing SEP changes during motor learning paradigms is a useful approach for identifying neurophysiological indicators of plasticity that occur as a result of learning-related sensorimotor reorganization and integration (Schwenkreis et al., 2001; Pleger et al., 2003; Macerollo et al., 2018; Ohashi et al., 2019). For example, previous motor learning studies consistently demonstrated functional neuroplasticity of the sensorimotor system indicated by SEP changes following skill training of the upper extremity (Schwenkreis et al., 2001; Pleger et al., 2003; Andrew et al., 2015; O'Brien et al., 2020). Similarly, professional musicians such as violin players show an asymmetric enlargement of the left-hand representation in the sensorimotor cortex as measured by SEPs (Schwenkreis et al., 2007), further supporting the notion of use-dependent neuroplasticity after the extensive practice of specific motor skills. Thus, SEP could provide a valuable, accessible method to study and quantify training-induced neuroplasticity of the sensorimotor system of athletes. The training process of athletes is typically characterized by repetitive execution of sport-specific movements, resulting in fine-tuned motor control and superior processing of perceptional inputs (Nakata et al., 2010), which could translate into physiologically meaningful SEP-modulation compared to sedentary individuals.

This systematic review aims to shed light on the possibility of using SEP measurements to investigate exercise-induced functional neuroplasticity of the sensorimotor system by reviewing studies comparing SEP parameters between athletes and healthy controls not participating in organized sports or between cohorts of athletes from different sports. The unique requirements sports pose on athletes have been shown to elicit specific structural and functional neuroplasticity in the athlete's brain (Yarrow et al., 2009; Nakata et al., 2010). For example, parameters such as predominant movement patterns, temporal and spatial differences in required muscle activation, as well as single or combined limb use, could be reflected in specific changes in SEP parameters. With this approach, we intend to highlight the potential of SEPs to serve as a marker for superior somatosensory processing in high-level athletic performance.

## Materials and Methods

This systematic review was conducted following the guidelines and recommendations contained in the PRISMA 2020 statement (Page et al., 2021).

### Eligibility Criteria

Studies were deemed eligible for analysis according to the PICOS inclusion criteria (Methley et al., 2014) if they contained the following factors:

- Population: healthy male or female adult athletes (participating regularly in organized sport for at least 2 years before the experiment), free of injury or neuronal disease
- Intervention: measurement and comparison of SEP parameters (short- and long-latency SEP) between athletes and controls or between different athlete categories
- Control: age-matched healthy controls, not participating in organized sport or regular exercise
- Outcomes: latency and amplitude of SEP waves, HFO
- Study design: intervention or cross-sectional studies

Articles not meeting the inclusion criteria were excluded from this systematic review.

### Information Sources

A systematic literature search was performed by two independent researchers (TM, SH) in the electronic databases PubMed, Web of Science, and SPORTDiscus into all available sources with publication year until March 2021. The reference lists of the included studies were also scanned to generate a broader scope of the search. Only studies published in the English language were reviewed and included in the systematic review.

### Search Strategy

Searches were performed in PubMed (all fields), Web of Science (all fields), and SPORTDiscus (all fields) using the keywords “somatosensory evoked potentials” AND “athletes” OR “sports” OR “exercise.”

### Selection Process

**Figure 1:**
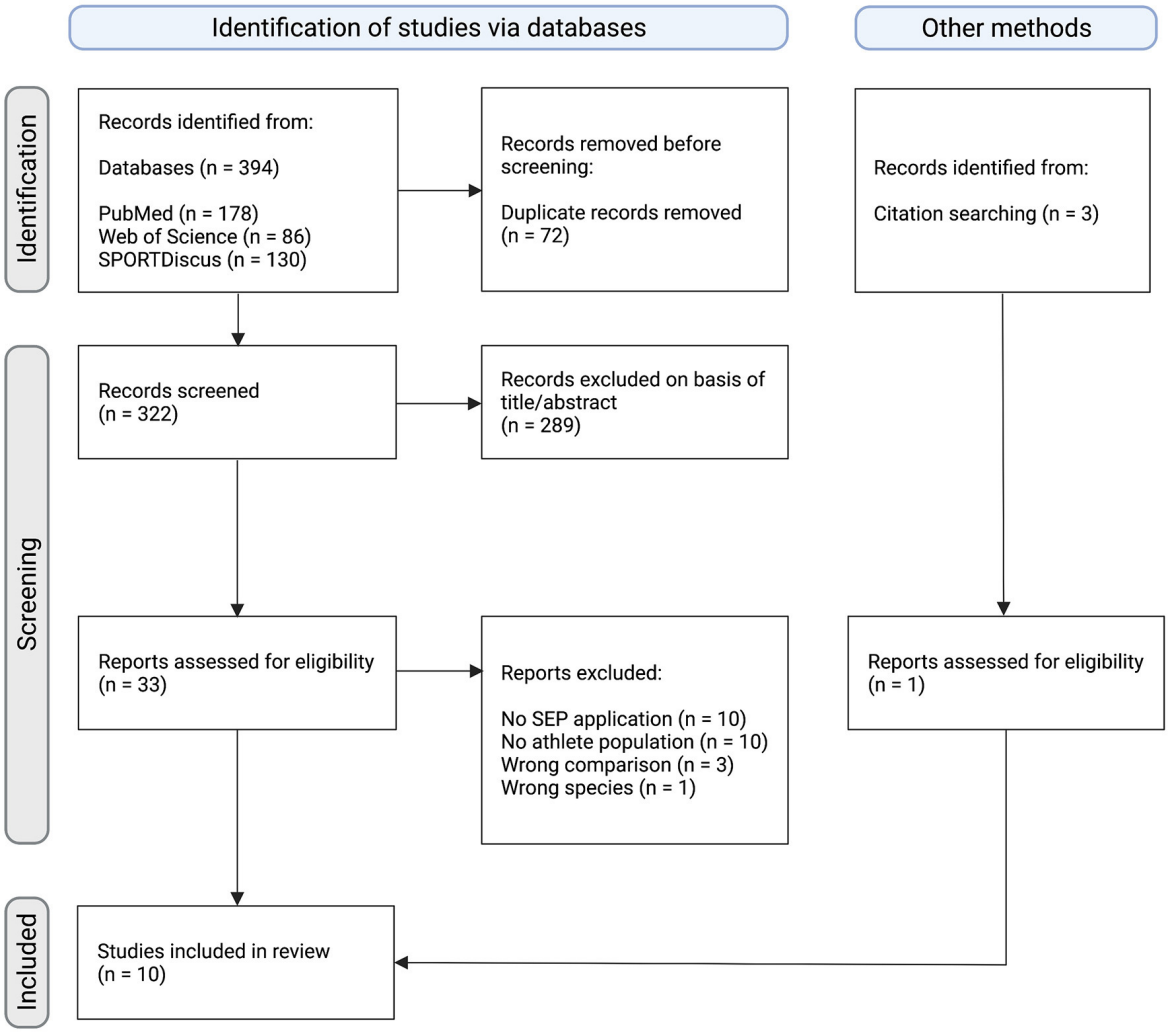
Flow chart diagram depicting the study selection process. Initially, 397 records were identified of which 10 studies were deemed eligible within the scope of the present systematic review. This figure was created with Biorender.com.

Records were screened and selected by two review authors (TM, SH) independently based on previously defined PICOS eligibility criteria (see flow diagram Figure 1). Disagreements were resolved by reaching a consensus or by involving a third person (PR).

### Data Collection Process and Data Items

Two review authors (TM, SH) independently collected the following data items from the included studies:

1. Methods: study design (cross-sectional, intervention)
2. Participants: number, mean age, age range, gender, inclusion criteria and exclusion criteria, sports discipline, history of sports participation.
3. SEP application: SEP stimulation sites (extremity and nerves), pulse characteristics, motor task during stimulation, and stimulation conditions.
4. Outcomes: primary and secondary SEP outcomes specified and collected, and time points reported.
5. Notes: funding for studies and notable conflicts of interest of authors.

Disagreements were resolved by reaching a consensus or by involving a third person (PR).

### Assessment of Methodological Quality

Assessment of methodological quality of eligible studies was performed by two review authors (TM, SH) independently using the JBI Critical Appraisal Checklist for Analytical Cross-Sectional Studies (Moola et al., 2020). This checklist consists of eight questions regarding the methodological quality of a study, that the reviewer has to answer with the following answers choices: Yes, No, Unclear or Not/Applicable (n.a.). Any disagreements in methodological quality were handled by a conversation between the two evaluators and consultation with the review's third author.

## Results

### Study Selection

The systematic literature search yielded a total of 394 records. After the removal of 72 duplicates, 322 records were screened, of which 289 were excluded based on title and abstract. The remaining 33 records were assessed for eligibility. Based on PICOS criteria, 24 records were excluded due to the following reasons: no SEP application (*n* = 10), no athlete population (*n* = 10), wrong comparison (*n* = 3), wrong species (*n* = 1). Furthermore, 1 study was identified through citation searching of screened studies. Finally, a total of 10 studies were deemed eligible for inclusion in this systematic review (Thomas and Mitchell, 1996; Kotzamanidis et al., 1997; Bulut et al., 2003; Iwadate et al., 2005; Murakami et al., 2008; Yamashiro et al., 2013, 2015, 2021; Enescu-Bieru et al., 2015, 2016). Overall, a total sample size of 231 participants was included. An overview of the study selection process is depicted in the following flow diagram (Figure 1) and characteristics of included studies are presented in Table 1.

**Table 1:** Overview of studies investigating athletes' SEP parameters.

**References**	**Design**	**Participants (F—female, M—male, TJ—training years of athletes)**	**Age (mean ±SD)**	**Stimulus (site, number of pulses, length, ISI)**	**Task during stimulation**	**Δ SEP amplitude**	**Δ SEP latency**	**SEP correlation (pos. —positive, neg. —negative)**
Thomas and Mitchell (1996)	Cross-Sectional	7 non-athletes (5F, 2M) 10 runners (4F, 6M) 7 elite gymnasts (1F, 6M) TJ: > 6 yrs.	22.1 ± 3.1 yrs. 28.4 ± 5.2 yrs. 21.0 ± 2.3 yrs.	Bilateral median nerve (wrist), 500 pulses, 0.2 ms, 0.5 s	None	None	None	Training years and the amplitude of N20 (pos.) Amplitudes of P11 and P13/14 and the number of hours of training per week (neg.) Amplitude of N30 and simple visual reaction time (pos.)
Kotzamanidis et al. (1997)	Cross-Sectional	14 non-athletes (all male) 14 weightlifters (n.a.) TJ: > 6 yrs.	22.0 ± 2.8 yrs. 24.0 ± 3.6 yrs.	Bilateral median nerve (wrist), 256–512 pulses, 0.2 ms, 2 s	None	None	n.a.	n.a.
Bulut et al. (2003)	Cross-Sectional/ Intervention	16 non-athletes (9F, 7M) 16 volleyball players (9F, 7 M) TJ: > 4 yrs.	20.6 ± 1.3 yrs. 20.7 ± 1.7 yrs.	Bilateral tibial nerve (medial malleolus), 250–500 pulses, 0.2 ms, 2 s	None	Right P60↓ in female athletes	Left P60↓ in male athletes	n.a.
Iwadate et al. (2005)	Cross-Sectional	7 non-athletes (all male) 7 football players (all male) TJ: n.a.	18.7 yrs. ± n.a. 21.8 yrs. ± n.a.	Left median nerve (wrist) Left Tibial nerve (ankle) 0.2 ms, 2–4 s	Oddball task	N140↑ in athletes (upper & lower limb) P300↑ in athletes (lower limb)	P300↓ in athletes (lower limb)	n.a.
Murakami et al. (2008)	Cross-Sectional	7 non-athletes (all male) 7 football players (all male) 7 racquet players (all male) TJ: > 4 yrs.	23.0 ± 1.9 yrs. 21.9 ± 1.1 yrs. 23.0 ± 2.1 yrs.	Bilateral median nerve (wrist) 2,500 pulses, 0.2 ms, 211–262 ms Bilateral tibial nerve (ankle) 2,500 stimuli,0.5 ms, 211–262 ms	None	P37–N45↑ in football players N20–P25↑ in racquet players	None	Starting age of training football and P37–N45 amplitude (neg.)
Yamashiro et al. (2013)	Cross-Sectional	15 baseball players (all male) 15 mixed athletes (2F, 13M) TJ: > 9 yrs.	20.3 ± 1.1 yrs. 21.7 ± 2.9 yrs.	Index finger (dominant hand) 140 pulses (70/70) 0.5 ms, 5–8 s	None Reaction time (RT) task	None	P100↓ & N140↓ in baseball players	RT and both the peak P100 and the peak N140 latencies (pos.)
Yamashiro et al. (2015)	Cross-Sectional	12 baseball players (all male) 12 mixed athletes (all male) TJ: > 9 yrs. / > 6 yrs.	21.2 ± 0.8 yrs. 22.7 ± 3.4 yrs.	Right index finger and pinky 400 pulses 0.2 ms, 2 s	GoNogo task	Nogo-N140↑ in baseball players	Nogo-N140↓ in baseball players	Nogo-N140 latency and GoNogo RT (pos.) Nogo-P140 amplitude and GoNogo RT (pos.) Nogo-P300 amplitude and GoNogo RT (neg.)
Enescu-Bieru et al. (2015)	Cross-Sectional	5 fencers (all male) 5 volleyball players (all male) 5 handball players (all male) TJ: > 5 yrs.	n.a. Range: 15–23 yrs.	Bilateral median nerve (wrist) 250–300 pulses 0.2 ms, 333 ms	None	None	None	n.a.
Enescu-Bieru et al. (2016)	Cross-Sectional	11 fencers (all male) 10 handball players (all male) TJ: > 6 yrs.	n.a.	Bilateral median nerve (wrist) n.a. 0.2 ms, 333 ms	None	None	None	n.a.
Yamashiro et al. (2021)	Cross-Sectional	10 baseball players (all male) 12 track & field athletes (all male) TJ: > 7 yrs.	21.5 ± 0.7 yrs. 20.2 ± 0.7 yrs.	Index finger and pinky (dominant hand) 100 pulses 0.2 ms, 2 s	GoNogo task	None	Go-P100↓ in baseball players Nogo-N140↓ in baseball players	Go-P100 latency and GoNogo RT (pos.) Nogo-P140 latency and GoNogo RT (pos.)

### Methodological Quality Assessment

**Figure 2:**
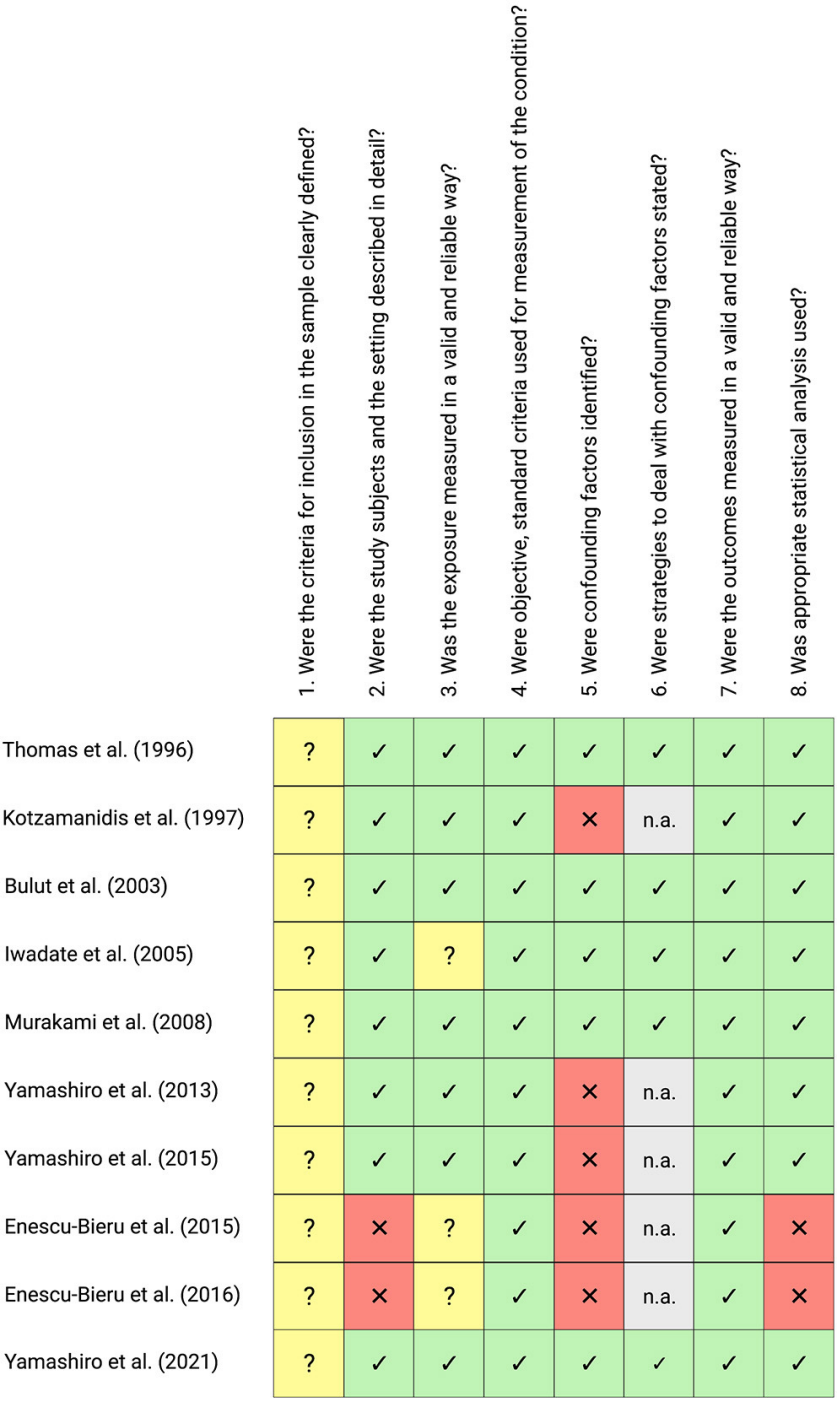
Methodological quality assessment of all included studies using the JBI Critical Appraisal Checklist for Analytical Cross-Sectional Studies. The checklist consists of eight questions regarding the methodological quality of a study, with the following choices: yes (green cells with ticks), no (red cells with crosses), unclear (yellow cells with question marks) or not/applicable (n.a., gray cells). This figure was created with Biorender.com.

The majority of the studies examined can be described as being of high methodological quality. However, heterogeneous results can be observed, with two studies showing considerable methodological problems due to incomplete specification of participant characteristics (i.e., no reports of anthropometric and demographic variables) and inappropriate statistical methods (e.g., uncorrected *post-hoc* comparisons). Furthermore, it is noteworthy that all studies examined provided incomplete information on the inclusion and exclusion criteria of the recruited participants (Question 1 of the JBI Checklist). Methodological quality assessment results were summarized and visualized in Figure 2.

### Study Design and Participant Characteristics

All of the included studies used a cross-sectional design, meaning athletes were compared cross-sectionally with control participants or other athletes of a different sport discipline (Thomas and Mitchell, 1996; Kotzamanidis et al., 1997; Bulut et al., 2003; Iwadate et al., 2005; Murakami et al., 2008; Yamashiro et al., 2013, 2015, 2021; Enescu-Bieru et al., 2015, 2016). Only one study additionally implemented a short-term intervention in form of an acute bout of exercising on a treadmill while comparing SEP parameters before and after the session (Bulut et al., 2003).

The majority of studies (seven out of 10) investigated exclusively male participants (Kotzamanidis et al., 1997; Iwadate et al., 2005; Murakami et al., 2008; Enescu-Bieru et al., 2015, 2016; Yamashiro et al., 2015, 2021). Furthermore, three studies investigated athletes and control participants with mixed-gender distribution (Thomas and Mitchell, 1996; Bulut et al., 2003; Yamashiro et al., 2013). Sample sizes were deemed small to moderate, ranging from five athletes per group (Enescu-Bieru et al., 2015) to 16 athletes per group (Bulut et al., 2003). Participants were mainly young adults in the age range of 18–34 years. One study included juvenile athletes as young as 15 years (Enescu-Bieru et al., 2015). In another study by the same leading authors the age of the participants was not reported (Enescu-Bieru et al., 2016). It should be noted that the majority of studies matched different groups of athletes or control participants for age (Bulut et al., 2003; Iwadate et al., 2005; Murakami et al., 2008; Yamashiro et al., 2013, 2015, 2021; Enescu-Bieru et al., 2016). This is an important prerequisite for group comparisons because age has a significant effect on the SEP according to IFCN guidelines (Nuwer et al., 1994). However, some studies either did not report that groups were age-matched (Kotzamanidis et al., 1997; Enescu-Bieru et al., 2015) or reported a slight but significant difference in age between groups (Thomas and Mitchell, 1996).

Athletes from the following sport disciplines were enrolled: running (Thomas and Mitchell, 1996), gymnastics (Thomas and Mitchell, 1996), weightlifting (Kotzamanidis et al., 1997), volleyball (Bulut et al., 2003; Enescu-Bieru et al., 2015), football (Iwadate et al., 2005; Murakami et al., 2008; Yamashiro et al., 2013), racquet sports (Murakami et al., 2008), baseball (Yamashiro et al., 2013, 2015, 2021), fencing (Enescu-Bieru et al., 2015, 2016), handball (Enescu-Bieru et al., 2015, 2016), track and field (Yamashiro et al., 2013, 2015, 2021), and swimming (Yamashiro et al., 2013, 2015). All athletes included in these studies trained regularly for at least 4 years in their respective sport at the time of the experiments. One study did not report the total amount of training years but the time of exhaustive exercise during an average week (9.85 h/week) over the previous 1-year period (Iwadate et al., 2005).

### Methodological Characteristics of SEP Measurements

Generally, methodological characteristics of SEP measurements were highly heterogeneous across studies. Important aspects regarding stimulation site, number of applied pulses, pulse length, pulse intensity, inter-stimulus interval, stimulation conditions, and signal recording are summarized below.

All studies investigating short-latency responses were additionally evaluated regarding adherence to IFCN guidelines (Nuwer et al., 1994; Cruccu et al., 2008). These guidelines propose the following stimulation and recording conditions:

Median nerve stimulation:

- Stimulation site: median nerve at the wrist
- Pulse length: 0.2 ms
- Pulse intensity: motor threshold (sufficient intensity to cause a thumb movement of 1–2 cm)
- Stimulus rate: 3–5 Hz
- Number of averaged pulses: 500 (1,000–2,000)
- Peripheral recording electrodes: Erb's point, C5 (skin over the spinous process)
- Cortical recording electrodes: Fz, C3'/C4' (10/20 system)
- SEP components: N9, N13, P14, N20, P20, N30

Tibial nerve stimulation:

- Stimulation site: tibial nerve at the ankle (medial malleolus)
- Pulse length: 0.2–0.3 ms
- Pulse intensity: motor threshold (sufficient intensity to cause a plantar flexion of 1–2 cm)
- Stimulus rate: 3–5 Hz
- Number of averaged pulses: 500 (1,000–2,000)
- Peripheral recording electrodes: popliteal fossa, L1 (skin over the spinous process)
- Cortical recording electrodes: Fz, Cz' (10/20 system)
- SEP components: N8, N22, P30, P39

**Figure 3:**
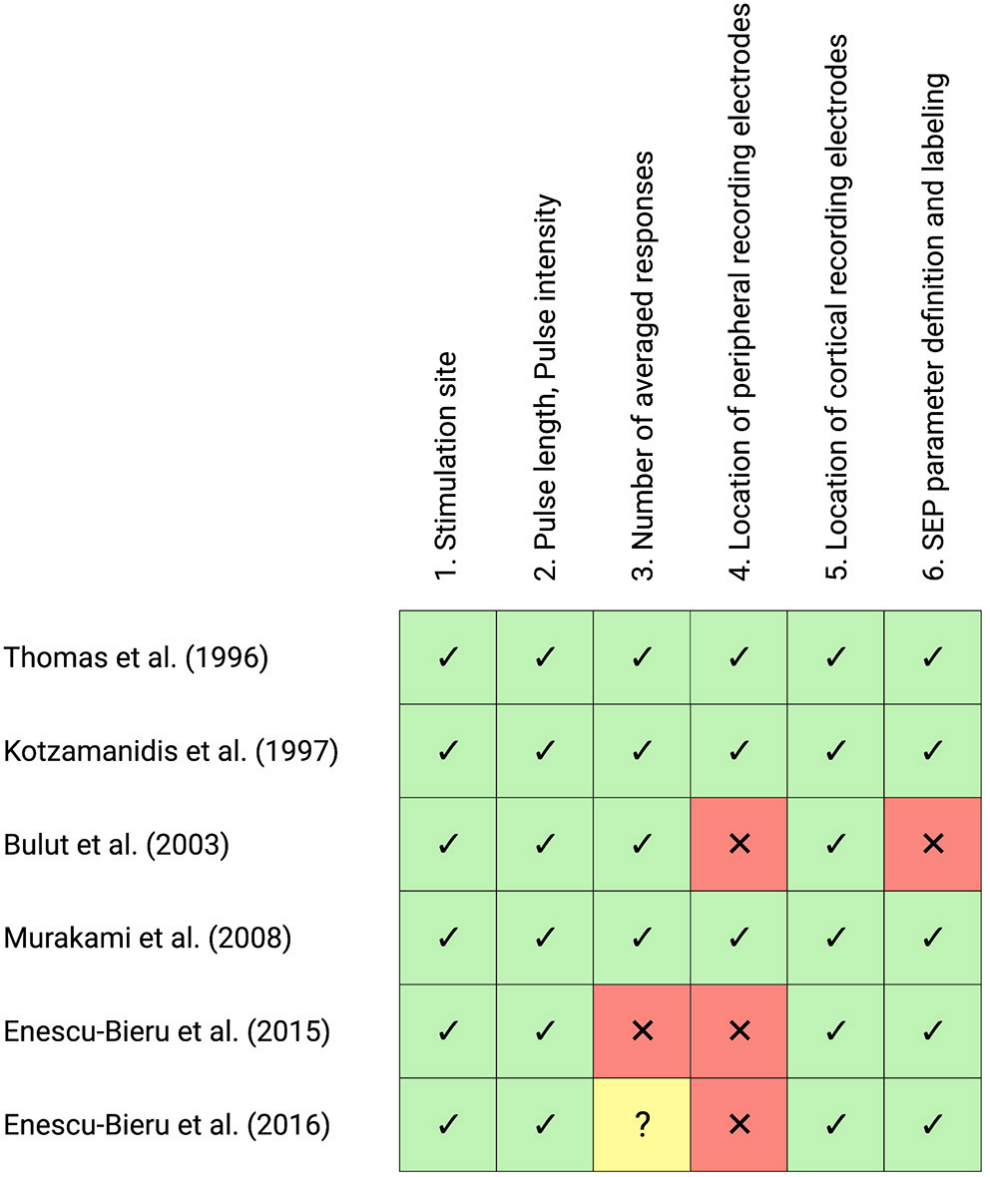
Evaluation of Somatosensory-evoked potential (SEP) methodology of all included studies investigating short-latency SEP according to IFCN guidelines. The checklist consists of seven items regarding the methodological recommendations of SEP measurements, with the following choices: agreement (green cells with ticks), violation (red cells with crosses), unclear (yellow cells with question marks). This figure was created with Biorender.com.

The result of this evaluation is visually summarized in Figure 3.

#### Stimulation Site

Stimulation sites of SEP measurements for upper and lower limb responses were evoked by either median nerve stimulation at the wrist (Thomas and Mitchell, 1996; Kotzamanidis et al., 1997; Iwadate et al., 2005; Murakami et al., 2008; Enescu-Bieru et al., 2015, 2016), median nerve stimulation at the index finger or pinky finger (Yamashiro et al., 2013, 2015, 2021), tibial nerve stimulation at the medial malleolus (Bulut et al., 2003) or tibial nerve stimulation at the ankle (Iwadate et al., 2005; Murakami et al., 2008).

#### Number of Pulses

The number of applied stimulation pulses during the experiment ranged from 100 (Yamashiro et al., 2021) to 2,500 (Murakami et al., 2008), with most studies using 250–500 pulses (Thomas and Mitchell, 1996; Kotzamanidis et al., 1997; Bulut et al., 2003; Enescu-Bieru et al., 2015). Two studies did not report the total number of stimulation pulses (Iwadate et al., 2005; Enescu-Bieru et al., 2016).

#### Pulse Length

Pulse length was generally 0.2 ms for median nerve stimulation (Thomas and Mitchell, 1996; Kotzamanidis et al., 1997; Iwadate et al., 2005; Murakami et al., 2008; Enescu-Bieru et al., 2015, 2016; Yamashiro et al., 2015, 2021). However, for tibial nerve stimulation, heterogeneous pulse lengths of 0.2 ms (Bulut et al., 2003; Iwadate et al., 2005) and 0.5 ms (Murakami et al., 2008) were applied.

#### Pulse Intensity

Intensities of constant current square wave pulses in most studies were directly at or slightly above the lowest intensity to consistently evoke twitching of the thumb, termed motor threshold (Thomas and Mitchell, 1996; Kotzamanidis et al., 1997; Bulut et al., 2003; Iwadate et al., 2005; Murakami et al., 2008; Enescu-Bieru et al., 2015, 2016). In some of the identified studies, weaker stimulus intensities that were directly at or up to three times the lowest intensity were used to produce a perceptual sensation, referred to as the sensory threshold (Iwadate et al., 2005; Yamashiro et al., 2013, 2015, 2021).

#### Inter-stimulus Interval

Inter-stimulus intervals (ISI) of peripheral nerve stimulations were fixed in some of the identified studies, i.e., at 333 ms (Enescu-Bieru et al., 2015, 2016), 0.5 s (Thomas and Mitchell, 1996), and 2 s (Kotzamanidis et al., 1997; Bulut et al., 2003; Yamashiro et al., 2015, 2021). However, variable and randomly jittered ISIs were used in others, i.e., 211–262 ms (Murakami et al., 2008), 2–4 s (Murakami et al., 2008), and 5–8 s (Yamashiro et al., 2013).

#### Stimulation Condition

The majority of studies assessed SEPs while participants were in a relaxed and passive state (supine position: *n* = 3; sitting position: *n* = 1; position not reported: *n* = 3) during application of the stimulation and signal recording (Thomas and Mitchell, 1996; Kotzamanidis et al., 1997; Bulut et al., 2003; Murakami et al., 2008; Yamashiro et al., 2013; Enescu-Bieru et al., 2015, 2016). One study implemented an oddball task by applying two different stimulation intensities, where participants had to press a button with the thumb of their right hand as quickly as possible whenever a deviant stimulus was presented (Iwadate et al., 2005). Similarly, another study asked participants to react as fast as possible by pressing a button with the index finger every time they perceived the somatosensory stimulation (Yamashiro et al., 2013). Furthermore, two studies used a Go-Nogo task where stimuli were applied either to the index finger of the participant (corresponding to the Nogo-condition) or to the fifth finger (corresponding to the Go-condition) (Yamashiro et al., 2015, 2021). Whenever participants perceived the stimulus at the fifth finger (Go), they were instructed to press a button as quickly as possible with the contralateral index finger. However, when participants perceived stimulation at the index finger (Nogo) they were instructed to suppress any response (Yamashiro et al., 2015, 2021).

#### Signal Recording

EEG was generally recorded using Ag-AgCl electrodes placed on the scalp corresponding to the international 10/20 system and with impedances kept below 5 kΩ. However, the number and placement of measuring electrodes differed between studies: two electrodes placed 1 cm behind C3/4 (Thomas and Mitchell, 1996), two electrodes placed on C3/C4 (Kotzamanidis et al., 1997), one electrode placed 2 cm behind the vertex (Bulut et al., 2003), five electrodes placed on Fz, Cz, Pz, C3 & C4 (Iwadate et al., 2005), foue electrodes placed on Cz' (2 cm posterior to Cz), C3' (2cm posterior to C3), C4' (2 cm posterior to C4) & Fz (Murakami et al., 2008), nine electrodes placed on F3/F4, C3/C4, P3/P4, Fz, Cz, Pz (Yamashiro et al., 2013, 2015, 2021) and 2 electrodes placed on C3' (2 cm posterior to C3) & C4' (2 cm posterior to C4) (Enescu-Bieru et al., 2015, 2016).

Additionally, two studies recorded peripherally evoked potentials by placing electrodes on Erb's point & the intervertebral space C6-C7 (Kotzamanidis et al., 1997) or Th12, Ic, C5s, Erb1 & Erb2 (Murakami et al., 2008). Moreover, further peripheral measures of nerve conduction velocity, distal sensory conduction time, and sensory nerve action potentials have been implemented to control for peripheral influences on SEP generation in some studies (Thomas and Mitchell, 1996; Kotzamanidis et al., 1997; Murakami et al., 2008).

### Main Results

Of the 10 included studies, six studies investigated short-latency SEP parameters (Thomas and Mitchell, 1996; Kotzamanidis et al., 1997; Bulut et al., 2003; Murakami et al., 2008; Enescu-Bieru et al., 2015, 2016) while four studies focused on the analysis of long-latency SEP parameters (Iwadate et al., 2005; Yamashiro et al., 2013, 2015, 2021). In addition, one study analyzed HFO (Murakami et al., 2008).

#### Short-Latency SEP

Two studies found differences in short-latency SEP parameters between athletes and healthy controls (Bulut et al., 2003; Murakami et al., 2008). Murakami et al. (2008) observed significantly larger bilateral N20-P25 amplitudes in racquet players and larger bilateral P37-N45 amplitudes in football players (Murakami et al., 2008) as compared to healthy controls. The authors speculated that the afferent input from long-term training in athletes may reorganize the somatosensory cortex of the trained limbs (i.e., upper limbs for racquet players and lower limbs for football players) (Murakami et al., 2008). Another study observed that right P60 amplitudes in female volleyball players and left P60 latencies in male volleyball players were significantly smaller compared to sedentary controls (Bulut et al., 2003). According to the authors, this result may suggest that regular exercise leads to attenuated SEP peak amplitudes (Bulut et al., 2003).

The remaining studies (*n* = 4) did not observe differences in short-latency SEP parameters between athletes and healthy controls or between athletes of different sports (Thomas and Mitchell, 1996; Kotzamanidis et al., 1997; Enescu-Bieru et al., 2015, 2016).

In addition to comparisons of SEP amplitudes and latencies, some studies performed correlation analyses (Thomas and Mitchell, 1996; Murakami et al., 2008). In this regard, Thomas and Mitchell (1996) found significant positive correlations between the number of training years and the amplitude of N20 as well as between simple visual reaction time and the amplitude of N30. Furthermore, they showed a significant negative association between both the amplitudes of P11 & P13/14 and the number of training hours per week (Thomas and Mitchell, 1996). Murakami et al. (2008) observed a significant negative correlation between the starting age of football training and the P37-N45 amplitude, indicating that the earlier an athlete started playing football, the larger the P37-N45 amplitude (Murakami et al., 2008).

#### Long-Latency SEP

Studies on long-latency SEPs (*n* = 4) also revealed differences between athletes and healthy control participants or between different athlete cohorts (Iwadate et al., 2005; Yamashiro et al., 2013, 2015, 2021).

Iwadate et al. (2005) found greater N140 amplitudes following upper and lower limb stimulation as well as greater P300 amplitudes after lower limb stimulation in soccer players. Further, they observed shorter P300 latencies after tibial nerve stimulation in soccer players, indicating superior somatosensory cognitive processing in athletes which require attention and skilled movements (Iwadate et al., 2005).

Yamashiro et al. (2013) reported shorter P100 and N140 latencies in baseball players in conjunction with shorter reaction times in a button press task compared to a group of athletes from different sports that did not require fine somatosensory discrimination and motor control of the hand. Additionally, they found that reaction times significantly correlated positively with both P100 and N140 peak latencies when baseball players and mixed athletes were pooled. These observations led the authors to conclude, that specific training of upper limbs may induce alterations in cortical areas involved in somatosensory processing and rapid initiation of motor responses (Yamashiro et al., 2013).

The same group of authors provided further evidence for superior sensorimotor inhibitory processes in baseball players compared to a group of athletes from different sports by observing greater N140 amplitudes in the frontal area and shorter N140 latencies during Nogo-trials of a GoNogo-reaction time task (Yamashiro et al., 2015). Moreover, they found that Nogo-N140 latencies significantly correlated positively with GoNogo reaction times. In addition, both Nogo-N140 and Nogo-P300 amplitudes significantly correlated with GoNogo reaction times, indicating that larger amplitudes of the Nogo-potentials led to shorter reaction times and therefore might reflect superior response inhibition (Yamashiro et al., 2015).

Another study by the same group of authors provided the first evidence that neuroplastic changes in athletes seem to be modality-specific (Yamashiro et al., 2021). They found that baseball players exhibit shorter P100 latencies during Go-trials and shorter N140 latencies during Nogo-trials of a GoNogo-reaction time task compared to a group consisting of track & field athletes. However, no differences between these groups were found concerning evoked responses obtained during an auditory GoNogo paradigm. Additionally, significant positive correlations between both the Go-P100 latencies as well as Nogo-N140 latencies and GoNogo reaction times were observed, while no such correlations were found for auditory responses (Yamashiro et al., 2021).

**Figure 4:**
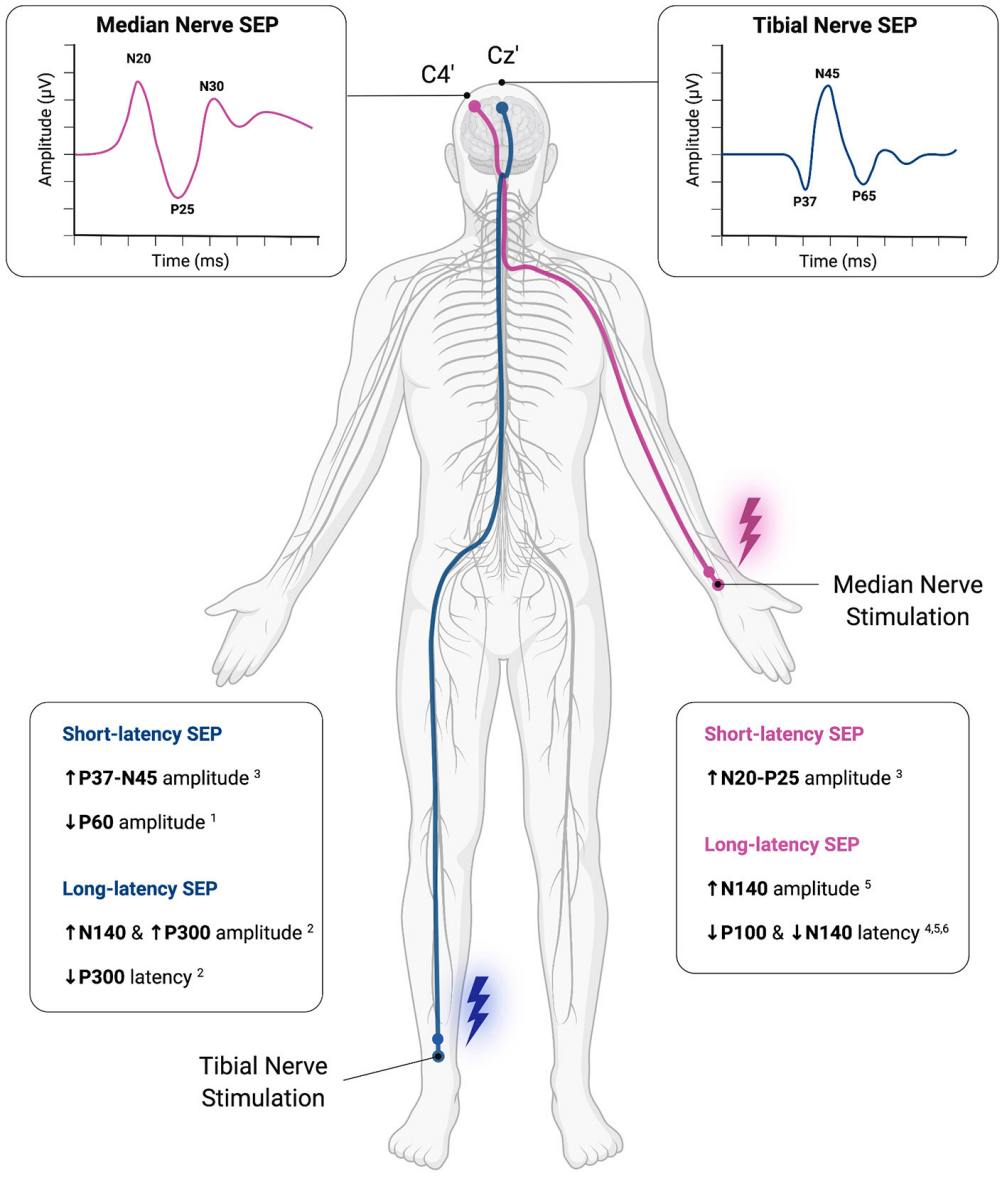
Methodological overview of somatosensory-evoked potential (SEP) application and summarized results of SEP modulations observed in athletes. Commonly, electrical stimulation of a peripheral nerve, i.e., median nerve (Violet) and tibial nerve (dark blue) is applied to examine upper and lower limb evoked responses, respectively. Evoked responses are recorded time-locked and averaged using EEG with different latencies from the onset of peripheral stimulation (e.g., N20, P37). Differences between athletes and healthy controls or between cohorts of athletes from different sports have been found for both short- and long-latency SEPs. These results suggest, that functional neuroplasticity within the somatosensory system in athletes may be identified via SEP recordings. This figure was created with Biorender.com. ^1^Bulut et al. (2003); ^2^Iwadate et al. (2005); ^3^Murakami et al. (2008); ^4^Yamashiro et al. (2013); ^5^Yamashiro et al. (2015); ^6^Yamashiro et al. (2021).

A summary of reported SEP modulations in athletes can be found in Figure 4.

#### High-Frequency Oscillations

With regards to HFO, it was observed in one study, that the number of negative peaks of HFOs following posterior tibial nerve stimulation in football players and the HFO amplitudes after median nerve stimulation in racquet players were significantly greater than those in non-athletes (Murakami et al., 2008). Therefore, the authors concluded, that the observed differences in HFO in athletes imply increased excitability in the somatosensory cortex of the skilled limb (i.e., leg & arm) induced by the respective training regimen (i.e., football vs. racquet sports).

## Discussion

Collectively, the available evidence reviewed here suggests, that functional neuroplasticity within the somatosensory system in athletes may be identified via SEP recordings. Differences between athletes and healthy controls or between cohorts of athletes from different sports have been found for both short- and long-latency SEPs. Amplitudes of short-latency responses were larger in athletes when stimulation of the median nerve or tibial nerve was applied (Murakami et al., 2008). Concerning long-latency SEPs, shorter latencies combined with increased SEP amplitudes were observed in soccer (Iwadate et al., 2005) and baseball players (Yamashiro et al., 2013, 2015, 2021), indicating superior somatosensory cognitive processing in sports that require high levels of attention, rapid initiation of motor responses, and response inhibition. However, other studies have failed to provide evidence of such SEP differences between athletes and healthy controls or athletes from different sports (Thomas and Mitchell, 1996; Kotzamanidis et al., 1997; Enescu-Bieru et al., 2015, 2016). The lack of significant results may be due to the respective sports and differences in training regimes examined in these studies, as weightlifting (Kotzamanidis et al., 1997), running, and gymnastics (Thomas and Mitchell, 1996) are predominantly determined by fundamental capabilities like strength, power, and endurance (Lee et al., 2020). On the other hand, sports such as volleyball (Bulut et al., 2003), football (Iwadate et al., 2005; Murakami et al., 2008) racquet sports (Murakami et al., 2008) and baseball (Yamashiro et al., 2013, 2015, 2021) place high demands on agility, coordination, and refinement of specific motor skills such as hitting, kicking, and passing. Accordingly, these more specific demands could elicit stronger, more unique changes in somatosensory networks that manifest in measurable SEP differences in these athletes. This is supported by the fact that neuroplastic changes in experienced athletes appear to be specific to the particular training regime and sport requirements, evidenced by differences in SEP parameters between different athlete populations following stimulation of the limb (arm or leg) that was primarily trained (racquet sports vs. football) (Murakami et al., 2008). Furthermore, using SEP measurements, baseball players were shown to exhibit shorter Go-P100 latencies and Nogo-N140 latencies elicited by somatosensory stimulation of their dominant hand in a GoNogo reaction time task compared to track and field athletes. However, no such difference in auditory evoked potentials was found in the same group of athletes. Therefore, the authors speculated that neuroplastic changes in athletes appear to be modality-specific, as baseball players showed better somatosensory processing and discrimination of digits (tactile modality) compared to track and field athletes, but no difference in auditory discrimination (auditory modality) (Yamashiro et al., 2021). Besides the type of sport, it is tempting to speculate that training status, i.e., competition level, and the total number of training years might play a role in the extent of SEP modulations observed in athletes. The development of complex sporting skills that correlate with structural and functional neuroplasticity in sensorimotor-related brain areas requires many years of deliberate practice (Yarrow et al., 2009). Accordingly, a certain amount of deliberate training has to be performed to elicit neuroplasticity in sensorimotor networks and the behavior of the athlete (Nakata et al., 2010). However, all studies included in this review, which reported training history, investigated athletes who had been training regularly in their respective sports for at least 4 years at the time of the experiments. Therefore, it seems unlikely that a low training status could have influenced the results reviewed here. Nevertheless, future studies comparing groups of athletes with different levels of expertise in the same sport, i.e., novices, intermediates, and experts, are needed to elucidate the temporal dynamics of training-induced neuroplasticity as assessed by SEP modulations.

Short-term intervention studies on the effects of acute exercise on SEP parameters suggest that moderate aerobic exercise can lead to functional neuroplasticity since modulations of short- and long-latency SEPs could be observed in previous investigations (Akatsuka et al., 2015; Perciavalle et al., 2015; Yamazaki et al., 2020). Therefore, it is reasonable to assume that similar findings could be observed for long-term exercise, which may lead to even more pronounced and chronically manifested functional neuroplasticity of the sensorimotor system, which can be captured by SEP measurements. Indirectly, this hypothesis is supported by correlational analyses performed in some of the studies reviewed. Specifically, one study found significant positive correlations between the number of training years and the N20 amplitude and between simple visual reaction time and the N30 amplitude (Thomas and Mitchell, 1996). Another study reported a significant negative correlation between the starting age of football training and the P37-N45 amplitude, implying that the earlier an athlete started playing football, the larger the P37-N45 amplitude (Murakami et al., 2008).

Interestingly, the number of negative peaks of high-frequency oscillations (HFO) appear to be enhanced in athletes following stimulation of the limb that was primarily trained (Murakami et al., 2008). Similar observations of enhanced HFO have been made in string players, presumably reflecting training-dependent cortical reorganization, more specifically the increase in synchronized activity of fast-spiking interneurons (Hashimoto et al., 2004). While the physiological underpinnings of HFO are still debated (Ozaki and Hashimoto, 2011), HFO might serve as another potential marker for superior sensorimotor processing in athletes and should be analyzed in future studies.

In practical terms, SEPs can be generated and recorded with relative ease using EEG electrodes. Furthermore, the technical prerequisites are inexpensive in comparison to other neuroimaging methods. EEG provides a higher temporal resolution and represents a more direct measure of neuronal activity compared to methods based on blood flow changes like fNIRS or fMRI (Macerollo et al., 2018). A limitation in SEP recordings is their low spatial resolution (Seidel-Marzi and Ragert, 2020). However, this depends on the EEG electrode montage used, i.e., single electrode recordings have a lower spatial resolution compared to whole-brain EEG configurations. Another drawback of SEP measurements is that SEPs can only be recorded in a stationary setting due to their high susceptibility to motion artifacts (Symeonidou et al., 2018), which limits its application in mobile sports settings. Still, another big advantage is that the SEP parameters themselves are robust and reliable. A recent multicenter investigation of common electrophysiological measures revealed high intraclass coefficients for SEP amplitudes at the motor threshold (ICC = 0.91) and N20 latencies (ICC = 0.90) following repeated measurements at baseline, 12, and 24 months follow-up (Brown et al., 2017). Thus, the requirements for a reliable diagnostic tool for the assessment of functional neuroplasticity in the sensorimotor system that can be used repeatedly for the quantification of training effects or differences between athlete groups seem to be met. However, latencies of the same SEP component depend on individual variation in limb length and general body size since conduction distances in afferent nerve pathways increase with increasing body proportions (Soudmand et al., 1982; Aminoff and Eisen, 2012). To resolve this confound, SEP results should be corrected for individual body proportions, as has been done previously in some studies (Thomas and Mitchell, 1996). In addition, peripherally attached electrodes, e.g., at the ear lobe (Ragert et al., 2011), Erb's point or in the intervertebral spaces (Kotzamanidis et al., 1997; Murakami et al., 2008), should be used to monitor segmental differences in somatosensory pathway conduction properties that could lead to observable SEP differences, regardless of cortical origin. In this regard, recommendations for peripheral electrode placements have been formulated in the clinical context (Nuwer et al., 1994; Cruccu et al., 2008). In this way, interindividual differences in SEPs can be identified with higher accuracy based on the underlying anatomical segment responsible for the observed differences/changes, i.e., cortical, subcortical, or spinal contributions. Finally, as proposed by other researchers, analysis of SEP parameters in both the time and frequency domains is needed to fully understand the location of their generation and the modulation of these generators by specific experimental procedures or interventions (Macerollo et al., 2018).

### Limitations and Recommendations for Future Studies

To some extent, inconsistencies in the methodology of SEP measurements (i.e., stimulus architecture, signal recording, task conditions, averaging of evoked responses) have been identified and may be responsible for heterogeneous results across studies. It is known that, for example, stimulus rates above 3 Hz can lead to peak attenuation i.e., a decrease in the amplitudes of peaks with more than 30 ms latency (Fujii et al., 1994). Especially for frontal peaks (N30b & P22), this can be observed. Accordingly, for certain peaks, there are recommendations to keep the stimulation rate below 3 Hz (Valeriani et al., 1998; Haavik and Murphy, 2013). An absence of differences in SEP amplitudes between athlete cohorts in some of the reviewed studies (Enescu-Bieru et al., 2015, 2016) could therefore also be explained by an inappropriate stimulus rate. However, it should be mentioned that changing the stimulus rate does not seem to affect the latency of the peaks (Fujii et al., 1994). Another important methodological point to consider is that SEP trials exceeding ± 20% of baseline values should be rejected for reasons of quality control. Additionally, latency values of separate trials should agree with each other to values below 0.25 ms to upper limb stimulation and 0.5 ms to lower limb stimulation (Nuwer et al., 1994; Mills, 2017). To address these issues, future studies should adopt a consistent SEP methodology (e.g., stimulus rate, trial rejection), following general recommendations formulated in the clinical context (Nuwer et al., 1994; Cruccu et al., 2008).

In addition, the comparison of SEP amplitudes between different individuals has some drawbacks which must be mentioned here. SEP amplitudes depend first of all on the spatial accuracy of recording electrodes. This spatial accuracy of the measurements should be ensured by using standardized EEG positions (e.g., international 10–10 or 10–20 system). Another limitation in deriving neurophysiological features from SEP signal amplitudes involves the fact that in group comparisons, differences in underlying tissue properties between groups potentially affect SEP amplitude. In longitudinal studies, this issue is addressed by normalizing SEP amplitudes to baseline values and then comparing changes in relative SEP amplitude between different groups (McGregor et al., 2015; Andrade et al., 2016; Anzellotti et al., 2016). However, in cross-sectional studies, this possibility does not exist. Due to differences in physiological composition between athletes and controls (e.g., potentially lower body fat in athletes), no definite conclusions can be drawn between signal amplitude and underlying neurophysiological characteristics, because the influence of tissue architecture on SEP amplitude is unclear. Future studies should consider normalizing recorded SEP amplitudes to parameters of the peripheral nerve volley, e.g., N9 potentials recorded over the brachial plexus (Nuwer et al., 1994) to refine conclusions about possible neuroplasticity. Similar approaches can be found in studies normalizing TMS parameters to M-waves (Turco and Nelson, 2021) or H-reflexes to M-waves (Grosprêtre and Martin, 2012). Furthermore, modeling studies should be undertaken to quantify and potentially eliminate the influence of tissue architecture on SEP amplitudes. Comparable approaches can be found in the field of transcranial direct current stimulation (Kalloch et al., 2020).

All studies examined were cross-sectional, which prevents causal inferences about the interplay between training-induced neuroplasticity in the somatosensory system and concomitant SEP modulations. The question of the relationship between nature and nurture thus remains unanswered, so the results reviewed here should be interpreted as preliminary and with caution. Crucially, future intervention studies in form of randomized controlled trials investigating different sports are needed to unravel the causal relationship between athletic training and specific SEP modulation as well as differential SEP modulations according to different training regimes.

Furthermore, the collective findings might not be sufficiently representative of both genders due to the overall small sample size and the even smaller number of female athletes included in the identified studies. In general, differences in brain structure and function between males and females have been revealed which might be reflected in differences at the behavioral level (Grabowska, 2017). Although initial results did not reveal sex differences in SEP measures when interindividual differences in limb length and body size were taken into account (Thomas and Mitchell, 1996), future studies should systematically investigate possible training-related differences between male and female athletes, with particular attention to the inclusion of more female athletes.

Finally, the small to moderate sample sizes in the studies reviewed here, ranging from 5 to 16 athletes per group, may be responsible for some apparent null findings. The problem of studies with insufficient sample size is a common challenge in sport and exercise science (Lohse et al., 2016) that needs to be addressed in future studies. Recommendations to improve power, precision, and sample size estimation in sport and exercise science research have recently been formulated and should be adopted (Abt et al., 2020).

## Conclusion

Based on the available evidence from cross-sectional studies, we propose that SEP measurements can be a valid and cost-effective neurodiagnostic tool for assessing functional neuroplasticity in the context of athletic performance. The potential of this method is supported by its ease of use and high inter-session reliability. Crucially, however, long-term interventions are required to validate the conclusions derived in this review that long-term athletic training leads to specific SEP modulations that are directly related to superior motor performance.

## Data Availability Statement

The original contributions presented in the study are included in the article/supplementary material, further inquiries can be directed to the corresponding author/s.

## Author Contributions

TM and SH independently performed the literature search. TM wrote the manuscript. SH, RK, and PR provided critical revision. All authors interpreted the data, contributed to the manuscript, reviewed, approved the content of the final version, and agree to be accountable for all aspects of the work.

## Funding

We acknowledge support from Leipzig University for Open Access Publishing.

## Conflict of Interest

The authors declare that the research was conducted in the absence of any commercial or financial relationships that could be construed as a potential conflict of interest.

## Publisher's Note

All claims expressed in this article are solely those of the authors and do not necessarily represent those of their affiliated organizations, or those of the publisher, the editors and the reviewers. Any product that may be evaluated in this article, or claim that may be made by its manufacturer, is not guaranteed or endorsed by the publisher.
